# Group-Based Consensus Scheme for Sensor-Event Consistency in Industrial IoT Environments

**DOI:** 10.3390/s26175611

**Published:** 2026-09-03

**Authors:** Soowang Lee, Seungbin Lee, Jiyoon Kim

**Affiliations:** 1 Department of AI Convergence Engineering, Gyeongsang National University, Jinju 52828, Republic of Korea; 2 Department of Computer Science and Engineering, Gyeongsang National University, Jinju 52828, Republic of Korea

**Keywords:** Industrial Internet of Things, practical Byzantine fault tolerance, consensus, sensor-event consistency

## Abstract

Industrial IoT (Internet of Things) systems increasingly depend on sensor reports for monitoring, automation, and operational decisions. However, sensor faults or Byzantine behavior can produce inconsistent or missing reports. PBFT (practical Byzantine fault tolerance) can maintain consistent processing among replicas despite a bounded number of Byzantine faults. Its overhead grows when many factory sensors participate in one expanding consensus group. Processing complete sensor-event records also increases local computation as record size grows. This paper proposes independent PBFT groups using fixed-length SHA-256 sensor-event digests. Groups are formed according to production processes or sensor characteristics. Each group limits consensus participation, while digests keep consensus-command size fixed. Raspberry Pi experiments separated grouping benefits from digest-processing benefits. Fixed-size groups moderated aggregate replica-local computation growth across 10–100 logical sensors. Digest processing became more beneficial as original sensor-event records increased in size. A four-device deployment also maintained consensus under evaluated Byzantine backup and primary faults. These results indicate that the scheme can reduce PBFT processing burden in resource-constrained IIoT deployments.

## 1. Introduction

With the expansion of Industry 4.0, manufacturing systems have evolved from isolated equipment automation to intelligent industrial environments where sensors, control devices, and computing infrastructure are interconnected. The Industrial Internet of Things (IIoT) is regarded as a key enabling technology for this transition. Through IIoT, physical equipment deployed in industrial sites is connected to the information and communication infrastructure. Consequently, equipment conditions and process-state data can be collected, exchanged, and analyzed in real time.

Among the components of IIoT systems, sensors are regarded as primary data-generating devices that connect physical processes with digital systems. Physical and operational conditions are detected by industrial sensors and converted into electrical or digital signals. The collected sensor data are transmitted to industrial gateways, edge servers, or control systems. They are then processed for equipment monitoring, process-state estimation, fault diagnosis, predictive maintenance, and automated control.

When the same equipment or interdependent processes are monitored by multiple sensors, their measurements can be combined to estimate the overall process state. Such a state may be difficult to determine from individual sensor data. Sensor data constitute essential information for industrial operation and control, and their accuracy and integrity directly affect the performance and safety of the entire IIoT system [1,2].

Sensor data collected and transmitted in smart factories may become inaccurate or unreliable due to non-malicious factors, such as sensor faults, system failures, and environmental conditions [3]. Malicious cyberattacks or intentional jamming can also compromise the reliability of data delivery [4]. Incorrect data or missing reports may be caused by sensor degradation, calibration errors, environmental interference, communication failures, and temporary hardware faults. Measurement consistency and data quality may be further degraded by unstable industrial operating conditions. In addition, heterogeneous sensors may differ in their communication standards, data formats, and reporting intervals. Consequently, data integration and interoperability problems may arise.

Sensor-data reliability can also be compromised when an attacker manipulates a sensor or its communication path [5]. Malicious manipulation of the communication path may cause information that differs from the actual process state to be delivered as a seemingly legitimate sensor report. Manipulated reports that preserve the statistical or temporal characteristics of legitimate data may not be identified by conventional validation and anomaly-detection mechanisms. As a result, system decisions may be distorted over an extended period. Sensor reports require validation across several complementary properties. These include temporal consistency, inter-sensor consistency, data provenance, and reporting-node trustworthiness. Such validation should not rely solely on predefined value ranges or single-point anomaly detection [6,7].

Malicious sensing participants that intentionally falsify their reports have been modeled as Byzantine attackers in recent studies [8,9]. Based on this model, a sensor that arbitrarily or maliciously deviates from normal reporting behavior is treated as a Byzantine replica in the proposed PBFT group. Multiple Byzantine replicas may also collude and submit consistent false reports, potentially distorting data-fusion results. Byzantine fault-tolerant consensus is used to maintain consistent processing results among non-faulty replicas.

Distributed state estimation based on multiple sensors can provide greater robustness and flexibility than single-sensor approaches. However, the increased exchange of information among sensors, gateways, and state-estimation nodes may also expand the attack surface. When both sensor measurements and intermediate information are manipulated, multiple nodes may produce mutually consistent results that do not reflect the actual process state. A mechanism based solely on node agreement may incorrectly accept manipulated results. The reliability of IIoT sensor data should be evaluated based on measurement anomalies, reporting-node trustworthiness, inter-sensor consistency, message integrity, and final-result consistency. These properties should be assessed collectively rather than through individual measurements or node agreement alone [10]. This paper focuses on replica-level processing consistency and authenticated consensus. Physical data accuracy, anomaly detection, and reputation assessment are outside its scope.

Prior studies have investigated anomaly detection and robust state estimation, reputation-based sensor evaluation, and Byzantine fault-tolerant consensus approaches. Anomaly detection and robust state estimation are used to identify abnormal data and estimate the system state under sensor faults. In reputation-based approaches, the influence of each sensor report is adjusted according to historical behavior and inter-sensor report consistency.

A consensus mechanism is still required to ensure that participating nodes reach a consistent processing result. Byzantine fault tolerance (BFT) consensus allows non-faulty replicas to reach agreement despite a bounded number of Byzantine replicas. Practical Byzantine fault tolerance (PBFT) can be applied when multiple sensor replicas observe the same or interrelated events. However, it requires repeated message exchanges, authentication, and verification. Both communication overhead and local computation must be considered when PBFT is executed on resource-constrained IIoT devices [11].

Not all smart-factory sensors monitor the same process or produce related data. Each production line has different monitoring targets and sensor configurations. Sensors producing the same data type can also form a separate group. Placing all sensors in one PBFT group enlarges that group as the factory expands. Each replica may then process more messages and authentication operations. Consensus scopes should follow actual production processes in smart factories.

Hierarchical and group-based architectures have been studied to reduce these costs. However, many existing approaches rely on gateways, committees, or upper-layer representatives, and their evaluations primarily examine latency, throughput, communication, or system-level scalability. In contrast, this paper compares a single-group baseline and the proposed group-based scheme through controlled measurements on the same target device [12,13,14].

The representation of consensus input can also affect replica-local computation. Using the complete sensor-event record increases input size with sensor data volume. This paper defines a fixed-length hash as a compact event digest. The hash is generated from a normalized sensor-event record. Using the digest keeps the consensus input size fixed as the original record grows. Section 3 describes the generation and verification of the compact event digest. To address these limitations, this paper proposes group-based PBFT using compact event digests. Sensors form independent replica groups according to production processes or sensor characteristics. Each replica compares the client-provided digest with its local digest before executing PBFT. Within the assumed Byzantine fault bound, correct replicas reach consistent processing results.

The main contributions of this paper are as follows. First, this paper proposes independent PBFT replica groups based on production processes or sensor characteristics. Each group uses a fixed-length digest of normalized sensor-event records as consensus input. Independent groups limit consensus participation, while digests keep input size fixed. Input size remains fixed regardless of the original record size. Second, this paper separates the effects of grouping and consensus input representation. It analyzes their effects on replica-local computation cost. Four-condition ablation measures changes across PBFT group sizes and original record sizes. For 10–100 logical nodes, this paper reports aggregate computation cost and steady-state PBFT message counts. Third, distributed operation is evaluated using an n=4, f=1 PBFT group on four Raspberry Pis. End-to-end latency and throughput are measured under normal and Byzantine backup fault conditions. For Byzantine primary faults, consensus recovery through VIEW_CHANGE and NEW_VIEW is verified.

The remainder of this paper is organized as follows. Section 2 reviews related work and the fundamental principles of PBFT. Section 3 describes the proposed architecture and consensus procedure. Section 4 presents the experimental methods and results. Section 5 and Section 6 discuss the implications, limitations, and future research directions.

## 2. Background and Recent Studies

Research on sensor-data integrity can be broadly categorized into anomaly detection and robust state estimation, reputation-based sensor evaluation, and blockchain-based consensus. Anomaly detection and robust state estimation are used to identify abnormal or manipulated data and estimate the system state despite faults or attacks affecting a subset of sensors. In reputation-based approaches, reporting-node trustworthiness is assessed based on historical behavior and inter-sensor report consistency. The resulting reputation scores are used to adjust the influence of sensor reports or determine eligibility for consensus participation [7,10]. Among blockchain-based consensus approaches, BFT protocols enable non-faulty nodes to reach a consistent result even when a bounded number of participating nodes behave arbitrarily. This section describes IIoT sensor-data reliability and the basic operation of PBFT. It also examines device constraints and reviews recent studies relevant to this paper. The approaches and evaluation scopes of related studies are compared to identify the research gap this paper addresses.

In IIoT systems, sensor data are used to support industrial operations and equipment condition monitoring. However, persistent security and management challenges can arise during sensor-data generation and processing. In next-generation industrial cyber–physical systems, sensors embedded in machines are integrated with cyber infrastructure and physical processes. Security mechanisms are required to protect both client- and server-side components of the overall system. In this context, sensor data comprise measurements collected from industrial equipment. These data support equipment monitoring and process control [15,16].

Threats to the reliability of sensor reports can be classified as non-malicious faults or intentional Byzantine behavior. Measurement noise, calibration errors, transient device failures, and communication faults may cause non-malicious sensors to produce inaccurate data or omit reports. In contrast, a Byzantine replica may omit reports, submit manipulated data as legitimate reports, or transmit conflicting values to different recipients. In this paper, reliability does not imply a guarantee that sensor data exactly match the actual physical state. Integrity means that unauthorized parties have not altered or forged sensor data. Consistency means that non-faulty replicas reach the same consensus result. Physical data accuracy is treated as a property distinct from integrity and consistency.

The participating nodes are sensors with local computation and communication capabilities. They share sensor data and exchange protocol messages with other sensors. Data from one sensor may not reveal faults or intentional manipulation. Several sensors must participate in consensus and reach the same result. The following subsection explains how PBFT supports this process under Byzantine faults.

### 2.1. PBFT and Byzantine Fault Tolerance

A node affected by a Byzantine fault may deviate arbitrarily from the prescribed protocol, for example, by omitting messages or sending conflicting values to different participants. PBFT employs state-machine replication to ensure that non-faulty replicas maintain a consistent request order and execution result despite such behavior. A system that tolerates up to *f* Byzantine replicas requires n≥3f+1 replicas. Within each view, one replica serves as the primary, while the remaining replicas serve as backups. The client submits an operation request, and the replicas determine the order of the request and execute it [11].

**Figure 1:**
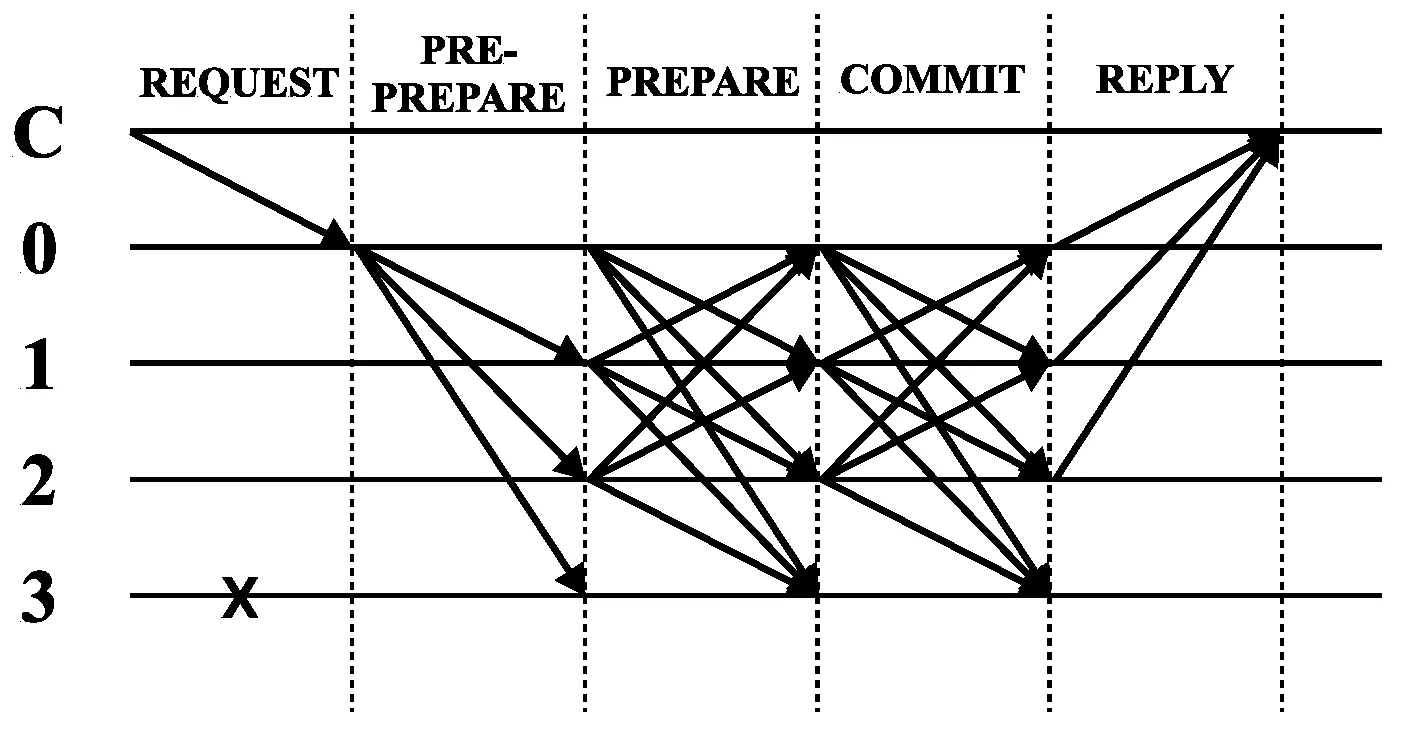
Message Flow of PBFT operation (with one faulty replica).

Figure 1 illustrates the message flow during PBFT operation. The client first sends a REQUEST to the primary. The primary assigns a sequence number to the request and multicasts a PRE-PREPARE message to the backup replicas. Upon accepting the PRE-PREPARE message, each backup multicasts a matching PREPARE message. Replicas that reach the prepared state then multicast COMMIT messages. Once the request is locally committed at a replica and all requests with lower sequence numbers have been executed, each non-faulty replica executes the operation and returns a REPLY directly to the client. In the figure, Replica 3 is designated as faulty and does not participate in the normal message flow. Arrows indicate message flows. An x marks the faulty replica. C denotes the client. Numbers 1–3 identify Replicas 1–3.

The PREPARE and COMMIT phases establish consistent evidence for the request digest and the sequence number assigned to the request. In the original PBFT protocol, a replica records the request and the corresponding PRE-PREPARE message and enters the prepared state after accepting 2f matching PREPARE messages sent by distinct backups. After reaching the prepared state, the replica enters the locally committed state only after accepting 2f+1 matching COMMIT messages from distinct replicas, which may include its own message. The client accepts the result after receiving f+1 replies from distinct replicas containing valid authentication information, the same request timestamp, and the same execution result. This client acceptance threshold is distinct from the replica commit quorum. These guarantees assume that protocol messages are authenticated. Digital signatures or message authentication codes are used to detect messages altered in transit and prevent attackers from impersonating correct replicas. Even in the presence of a tolerable number of Byzantine replicas, PBFT preserves the consistency of request ordering and execution results among correct replicas. However, it neither determines whether sensor data accurately represent the actual physical state nor detects every sensor failure. Each PBFT phase also involves local operations, including digest computation, message authentication, state updates, and message serialization. Deploying PBFT on sensor devices requires considering their limited resources.

PBFT initiates a view change if the primary provides no valid PRE-PREPARE. It also starts when consensus makes no progress for a predefined period. Backups send VIEW_CHANGE messages containing the current view’s progress state. The next primary collects the required VIEW_CHANGE messages and constructs NEW_VIEW. It safely reproposes requests unfinished in the previous view. Replicas resume PRE-PREPARE, PREPARE, and COMMIT in the new view. This procedure prevents a Byzantine primary from continuously delaying consensus. It also preserves request ordering across views.

PBFT assumes identity authentication for consensus participants and integrity protection for protocol messages. Cross-layer authentication combining physical- and application-layer characteristics can strengthen participating-device trustworthiness and message authentication [17]. However, authenticated participants can still omit messages or transmit conflicting information. Byzantine fault-tolerant consensus is needed alongside authentication to reach consistent processing results. This requirement allows such Byzantine behavior without compromising agreement. On resource-constrained devices, both functions must consider computational and memory overhead.

Sensor and edge devices provide local computation and communication capabilities but may have more limited CPU performance, memory, storage, and energy resources than server-class systems. These resource differences directly affect the implementation and evaluation of BFT protocols. In PBFT, each replica must generate and verify messages at each protocol phase, compute request digests and authentication information, and update the protocol state. As the number of participating replicas increases, the number of peer messages and authentication operations processed by each node may also increase. Both communication complexity and local computational burden must be considered. Throughput and consensus latency measured in blockchain or server-class environments may reflect the combined effects of hardware performance, network configuration, and workload characteristics. Consequently, these metrics do not isolate the computational effect of PBFT deployment choices. Evaluating PBFT in sensor environments requires separating local computation from network delivery and waiting time. Controlled measurements on the same target device provide a clearer comparison. Considering these constraints, the following subsection reviews existing studies that implemented BFT on embedded devices or adjusted the scope of consensus through grouping, hierarchical organization, and participant selection.

### 2.2. Classification of Recent BFT Studies

**Figure 2:**
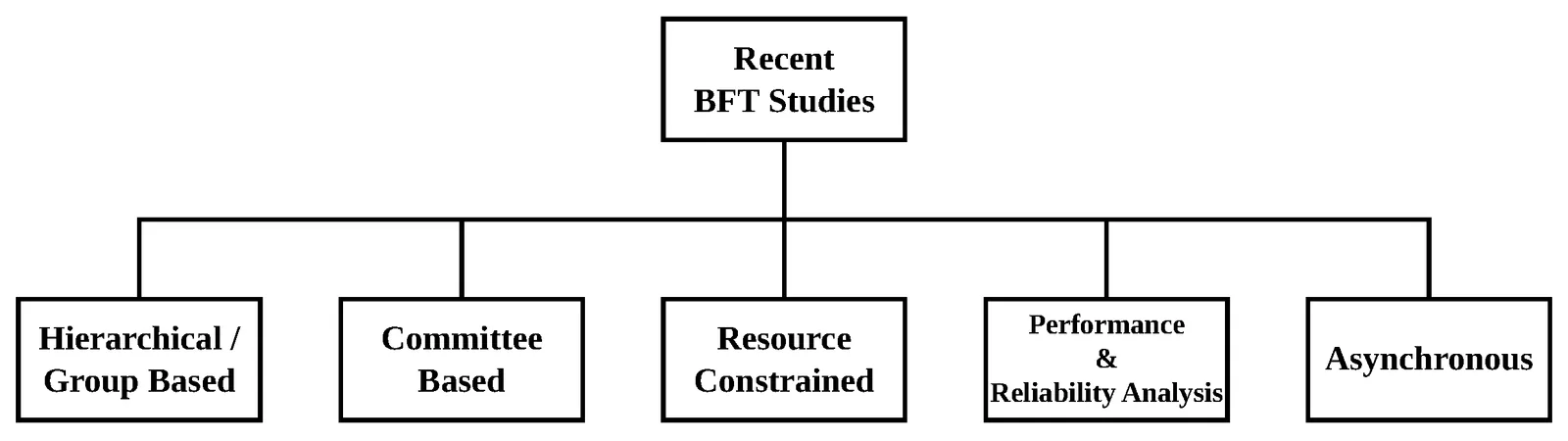
Taxonomy of recent BFT studies.

Zhong et al. [18] classified BFT algorithms by their primary performance-improvement methods. Their taxonomy covers grouping, multiple leaders, speculative execution, trusted hardware, and other approaches. Following this principle, this paper classifies eight studies by their main research focus. The categories reflect each study’s objective, design, and evaluation scope. Some studies relate to multiple categories. This paper assigns them according to their primary contribution. Figure 2 shows the taxonomy of the recent BFT studies.

- **Hierarchical/Group-Based BFT**: This category divides replicas by role, domain, process, or location. Consensus proceeds within groups and may continue through leaders or upper layers. This structure reduces direct communication across the complete replica network. It can also distribute consensus processing across several groups. Group formation and inter-group coordination influence performance, scalability, and Byzantine fault tolerance.
- **Committee-Based BFT**: This category selects a subset of nodes for each consensus round. Selection may consider reputation, resources, randomness, or current network conditions. A smaller committee reduces voting traffic and limits active consensus participants. Committee selection must still preserve Byzantine fault tolerance and fair participation.
- **Resource-Constrained BFT**: This category examines consensus execution on hardware with limited computing capacity. Such devices may also have restricted memory, energy, storage, and bandwidth. These studies optimize protocol state, allocation, batching, and message processing. Physical embedded platforms commonly evaluate latency, throughput, memory, and energy use. Their main concern is practical BFT operation under tight resource limits.
- **Performance and Reliability Analysis**: This category analyzes BFT using models, simulations, or networked experiments. It examines throughput, latency, communication, availability, node failures, and recovery. These studies reveal how scale and network conditions affect BFT operation. They usually emphasize system-level behavior rather than individual-device processing.
- **Asynchronous BFT**: This category removes fixed message-delivery assumptions from the consensus model. It supports progress under unpredictable communication delays and varying network conditions. Such protocols use reliable broadcast, threshold cryptography, ordering, or recovery mechanisms. Evaluations commonly examine throughput, latency, and communication under delayed networks.

Hierarchical/Group-Based BFT and Committee-Based BFT both adjust the scope of consensus participation. However, they organize participating nodes differently. Hierarchical/Group-Based BFT divides all replicas into multiple groups or layers. Consensus then proceeds within groups or across layers. Committee-Based BFT selects a subset of nodes from the entire network. Only selected nodes participate in a given consensus process. The first category partitions the consensus structure. The second category selects consensus participants.

### 2.3. Recent Studies

Research on scalable BFT has commonly reorganized replicas into hierarchical or group-based structures. These structures reduce communication between all replicas and distribute consensus processing. Table 1 compares the eight recent studies across all five research categories.

Guo et al. [12] proposed DCBFT for large IoT consensus networks. The protocol partitions proxy devices into domains using improved k-sums. It selects consensus nodes according to reputation and computing capacity. Multiple primary nodes coordinate consensus across two hierarchical levels. A dynamic reputation model rotates participating nodes after each round. The authors simulated 200 proxy devices on one Windows system. They evaluated communication consumption, throughput, latency, stability, and bandwidth requirements. DCBFT reduced communication growth and improved throughput in the reported simulations. This study did not use physical IoT devices.

Liu et al. [13] proposed Group BFT for scaling leader-based consensus systems. The protocol divides replicas into equal-sized groups. Each group leader aggregates member votes during the first round. It forwards an aggregated vote to the consensus leader during the second round. Standard and dynamic variants support fixed or exchanged group membership. The authors established safety, liveness, and responsiveness. Experiments covered 16 to 256 replicas on distributed cloud instances. Tests varied payload size, batch size, network delay, and Byzantine failures. Group BFT reduced leader bottlenecks and improved throughput at larger scales. The evaluation used cloud instances rather than physical IoT devices.

Li et al. [14] proposed a multi-layer PBFT architecture for large blockchain networks. The double-layer model contains Z=1+m+mn total nodes. The first-layer leader controls *m* replicas. Each first-layer replica leads *n* replicas in the second layer. These leaders collect member replies before returning aggregated results. The authors analyzed communication complexity and fault thresholds. Simulation-based validation used m=n=30. Separate analyses used m=n=500 and a 1000-node communication example. These cases represented different conditions rather than one experiment. The evaluation relied on analytical results and simulation-based validation without peer-to-peer execution.

Wu et al. [19] proposed DBPBFT for heterogeneous IoT data. It separates mining and packaging functions across two blockchains. The mining chain groups nodes according to data type. Reputation scores guide primary selection within each group. The packaging chain aggregates and verifies blocks from mining groups. Simulations varied system size from 200 to 1200 nodes. The authors recommended 10 to 20 nodes within each mining group. DBPBFT achieved lower consensus time and higher throughput than PBFT. This study did not report its execution platform or physical network configuration.

Together, these studies show that grouping can reduce communication and distribute consensus work. Many designs forward group results to an upper layer or global consensus. Most evaluations used simulations, servers, networked testbeds, or analytical models.

Committee-based BFT studies reduce consensus participation by selecting a subset of nodes. Selection criteria include location, reputation, network conditions, and learned system policies.

Kumar et al. [20] proposed R-PBFT for vehicular blockchain systems. It uses reputation to select a leader and N/3 secondary nodes. Digital signatures authenticate messages exchanged during consensus. The physical testbed used five Raspberry Pi 4 vehicle nodes. It also used two Jetson Nano edge servers. Experiments varied block sizes up to 1000 KB and processed 20,000 transactions. The evaluation covered delay, transaction frequency, throughput, congestion, and fairness. The authors reported 50% lower delay and 50% higher throughput. The evaluation used one vehicular testbed configuration.

This study reduces participation through reputation-based node selection. It can lower communication and consensus time in large networks. Its evaluation emphasizes system performance and the selection policy.

Resource-constrained BFT research examines whether consensus protocols can operate on limited hardware. These studies emphasize memory usage, processing capacity, and network-inclusive performance.

Böhm et al. [21] proposed TinyBFT for highly constrained embedded systems. TinyBFT implements PBFT-based state-machine replication with bounded memory usage. Static allocation and bounded protocol state limit runtime memory demand. Batching and hash-based ordering further reduce protocol overhead. The physical testbed contained four ESP32-C3 replicas. Each device provided a 160 MHz processor and 400 KB of memory. The replicas communicated through a 2.4 GHz Wi-Fi network. Experiments used small commands, 1 KiB operations, and Yahoo! Cloud Serving Benchmark (YCSB) workloads. The evaluation measured latency, throughput, memory consumption, and approximate energy demand. Small commands required approximately 50 ms, while large commands required approximately 80 ms. Throughput reached 31 writes and 43 reads per second. The initialized memory footprint was 165,515 bytes. These results demonstrate PBFT execution on constrained physical hardware. However, the reported latency included network communication among four replicas.

Both TinyBFT and this paper reduce PBFT overhead in resource-constrained environments. However, they target different design objectives. TinyBFT limits memory usage and protocol overhead through static memory allocation. It also uses bounded protocol state and hash-based ordering. By contrast, this paper limits consensus participation through independent replica groups. It fixes consensus input size using a compact event digest. In TinyBFT’s hash-based ordering, replicas disseminate full requests, and the primary proposes request hashes. In this paper, the event digest itself serves as the consensus command.

Performance and reliability studies analyze BFT through network and probabilistic models. These models examine scalability, queueing, network delays, failures, repairs, and system capacity.

Mišić et al. [22] proposed an adapted PBFT ordering service for blockchain-enabled IoT systems. Multiple orderers can receive and process IoT transaction batches. The model combines PBFT processing with bandwidth reservation and queueing. Maple calculations were validated through custom AnyLogic simulations. The evaluation used 4, 7, and 10 orderers. These configurations tolerate 1, 2, and 3 faulty orderers, respectively. Each request had a fixed 1 KiB size and contained 5 to 10 transactions. Network models included 2 Mbps links and several geographic delay conditions. The analytical and simulation results differed by approximately 1 to 2.5 percent. Small-area clusters provided about 60 percent more capacity than medium-area clusters. The evaluation relied on analytical modeling and simulation without physical execution.

This study clarifies how network conditions affect PBFT ordering performance. However, it relies on analytical modeling and simulation without physical execution.

Asynchronous BFT research removes timing assumptions that require timely message delivery. This approach supports consensus under unpredictable delays but requires additional coordination.

Liu et al. [23] proposed TortoiseBFT for asynchronous IoT blockchain systems. It applies an order-first design for asynchronous transaction consensus. A two-phase recovery process handles transactions missing from proposed blocks. Threshold signatures reduce repeated verification across asynchronous agreement procedures. A reputation model evaluates block producers during repeated consensus rounds. The testbed used three Dell R740 servers. It deployed 100 VirtualBox virtual machines across these servers. Each virtual machine used one processor core and 2 GB of memory. Experiments varied participating nodes from 4 to 100. Network delays ranged from 1 to 50 ms. Each transaction contained 250 bytes. Proposal sizes ranged from 256 to 16,384 transactions per node. The evaluation measured communication overhead, throughput, transaction latency, and agreement executions. TortoiseBFT reported about half the communication overhead of three asynchronous BFT baselines. It also achieved higher throughput and lower transaction latency. However, the testbed used server-based virtual machines rather than physical IoT devices.

The eight selected studies span hierarchical/group-based, committee-based, resource-constrained, performance and reliability, and asynchronous BFT. This paper separately analyzes independent PBFT groups and fixed-length event digests. It also evaluates computation costs, Byzantine fault handling, and view-change recovery.

## 3. Proposed Scheme

This paper proposes a group-based consensus scheme for consistent processing of IIoT sensor-event records. A sensor-event record contains the sensor data organized for one consensus round. Its compact event digest represents that record during consensus. Each replica group independently executes PBFT using this digest as the consensus command.

### 3.1. System Architecture

Sensors observing the same process line or generating correlated data form a PBFT replica group. A factory may contain multiple replica groups for different processes or sensor characteristics.

**Figure 3:**
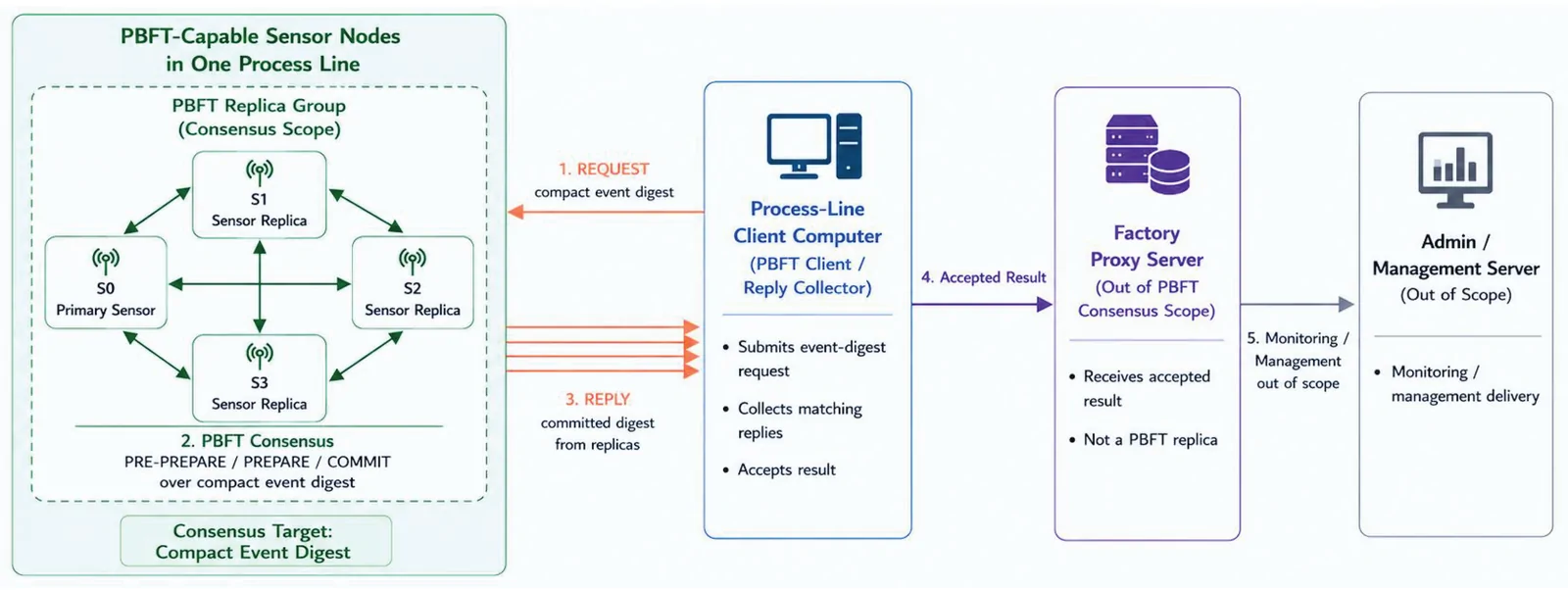
Architecture of the proposed group-based consensus scheme.

Figure 3 illustrates the components and message-flow boundaries of the proposed scheme. The architecture comprises sensors, a client, a factory proxy server, and a management server. In this example, four sensors, S0–S3, form one replica group. S0 serves as the primary, while S1–S3 serve as backups. This example uses n=4. The logical group size may vary across deployment environments. Steps 1–3 represent the client’s consensus request, replica-group consensus, and result return. Steps 4–5 show the accepted sensor data and consensus results reaching the factory proxy server. The proxy then forwards them to the management server.

The proposed scheme avoids placing all factory sensors in one large replica group. It creates multiple groups based on process or sensor characteristics. For example, sensors deployed within one production process can form a group. Pressure sensors and temperature sensors can also form separate groups. Each replica group performs PBFT consensus independently from other groups. Each group client forwards accepted sensor data and consensus results to the factory proxy server. A new production line can use a separate group without enlarging existing groups. Each group’s size can match its deployment environment. Consensus delays or failures within one group do not directly affect another group’s PBFT progress. This scheme separates group-level PBFT consensus from factory-level result collection.

**Figure 4:**
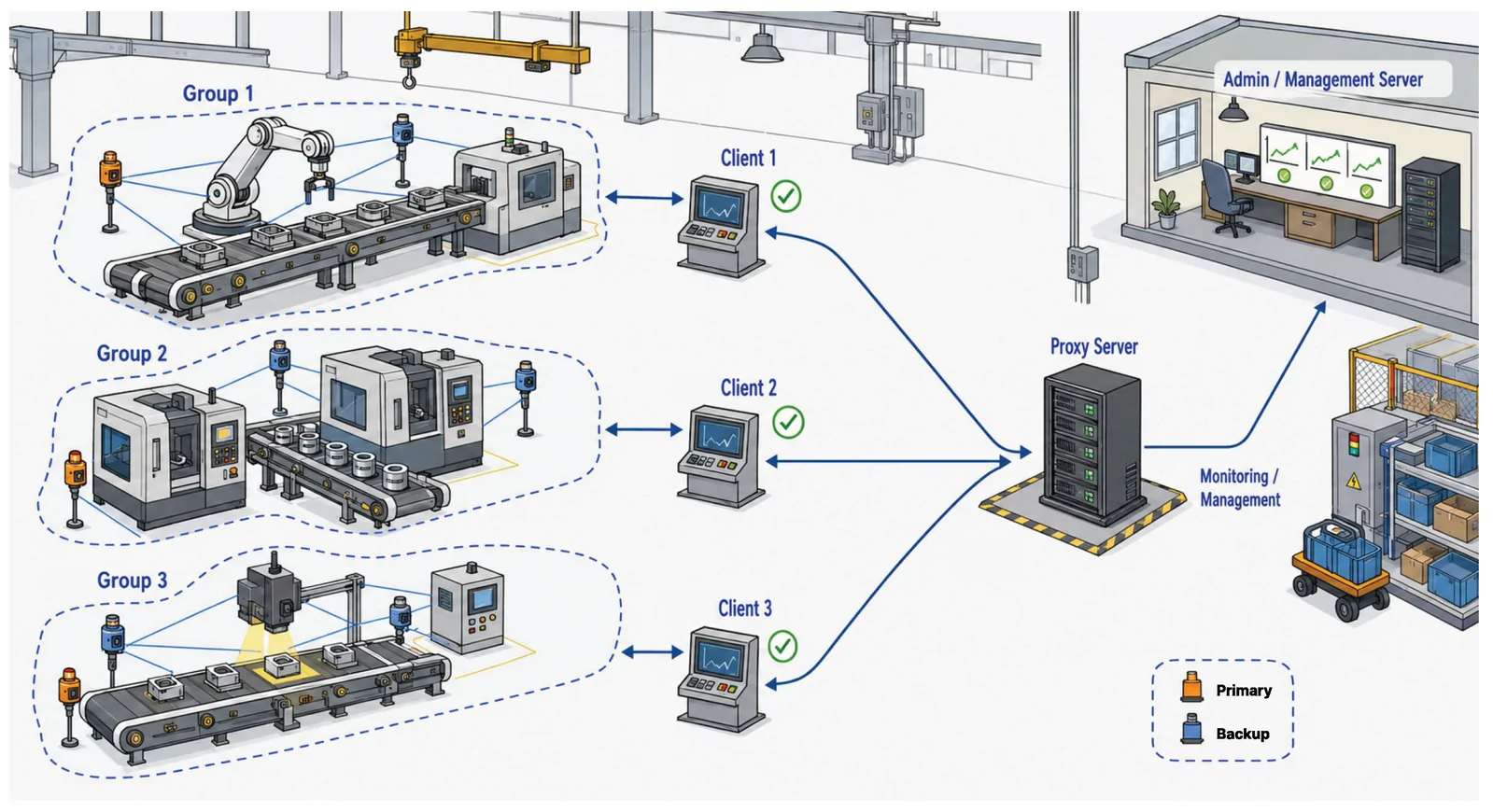
Illustration of smart factory scenario with group-based consensus scheme.

Figure 4 shows an example deployment of the proposed scheme in a smart factory scenario. Each dashed boundary represents a replica group for one production line or sensor set. The orange sensor serves as the primary, while blue sensors serve as backups. The client requests consensus from its replica group and collects the returned replies. It does not participate in consensus as a replica. The factory proxy server collects accepted results from multiple clients. It forwards these results to the management server. Subsequent processing by the management server is outside the scope of this paper.

Grouping sensors with related data or operational contexts allows their reports to be processed within a common scope. Previous work on IIoT data aggregation has shown that correlation-based clustering can organize sensor data into cohesive groups while reducing the influence of irrelevant or disparate data, thereby supporting reliable aggregation and subsequent decision-making [24]. In the proposed scheme, this concept is applied to consensus by allowing each sensor group to execute PBFT independently. The grouping does not improve the physical accuracy of individual sensor data; instead, it limits validation and consensus to sensors associated with the same process context, improving the consistency and efficiency of sensor-event processing.

### 3.2. Threat Model

The threat model follows the authenticated Byzantine fault model of PBFT. Within each replica group, up to *f* of the *n* sensor replicas may exhibit Byzantine behavior. A Byzantine replica may be either a primary replica or a backup replica, and arbitrary deviations from the prescribed protocol may occur. Manipulated sensor data may be provided, protocol messages may be omitted, or inconsistent information may be transmitted to different participants. Collusion among multiple Byzantine replicas within the same group is also permitted, and their behavior may be coordinated. The fault bound *f* is applied independently to each replica group.

In the current threat model, the client, factory proxy server, and management server are assumed not to exhibit Byzantine behavior. A consensus request is constructed and submitted by the client, and the result is accepted according to the defined reply threshold. The factory proxy server and management server are operated outside the PBFT replica group, and only results that have already been accepted by the corresponding client are processed. Byzantine fault tolerance for these components and compromise across different groups are outside the scope of this paper.

Communication follows the authenticated-message assumption of PBFT and is assumed to be conducted over secure communication channels. Pre-consensus payloads and protocol messages exchanged between the client and replicas, as well as among replicas, are transmitted through authenticated and encrypted channels. The identity of the sender and the integrity of each message are verified by the receiver before the message is processed. Messages transmitted over the network may be observed, delayed, dropped, duplicated, or reordered by an adversary. However, a valid authenticated message cannot be generated by impersonating a correct participant, and protected payloads cannot be recovered from ciphertext transmitted over the communication channel. Authentication and encryption keys are assumed to be securely established before protocol execution. Key establishment, key compromise, and key-recovery mechanisms are outside the scope of this paper.

Byzantine fault tolerance is provided by the proposed scheme only within each independent replica group. The objective of the proposed scheme is to preserve the integrity of sensor-event records and processing consistency among correct replicas within the assumed fault-tolerance bound. The physical accuracy of sensor data is not guaranteed, and Byzantine fault tolerance or consistency across different replica groups is not provided.

### 3.3. Proposed Group-Based Consensus Scheme

This paper assumes that sensors within the same replica group share their data through bidirectional communication before consensus. Each sensor also sends its data to the client, so the sensors and the client hold the same data when consensus begins. This pre-consensus sharing separates sensor-data acquisition from PBFT execution. PBFT is therefore performed on a representation of the sensor-event record already held by the participants rather than being used to distribute the original sensor data. Because differences in format or ordering can produce different digests even for the same sensor data, all participants organize the shared data using a predefined format and order.

All participants normalize sensor-event records using the same rules. Each sensor uses a unique identifier within its group. Timestamps are converted to a common time reference and predefined resolution. Fields are serialized in a predefined order. Missing values are recorded using explicit markers without estimation. Sensors with different sampling periods preserve measurements from the same event interval in timestamp order. No forced interpolation or resampling is performed. Heterogeneous sensor data are converted to a common schema. The schema defines data types, field representations, and units. These rules produce identical byte sequences for logically identical sensor-event records before hashing.

The predefined organization procedure ensures that logically identical sensor-event records are represented by the same byte sequence before hashing. Without this step, differences in field ordering or serialization could lead to different digest values even when participants hold the same sensor data. SHA-256 was selected as the compact event-digest function because it is a standardized SHA-2 algorithm and produces a fixed 256-bit digest [25]. SHA-256 has also been adopted as a cryptographic hash function in recent PBFT-based systems, including PBFT schemes for IoT and blockchain environments [26]. Compared with SHA-512, SHA-256 provides a shorter 32-byte digest rather than a 64-byte digest. Since the digest in the proposed scheme serves as a compact representation of a sensor-event record, the additional digest length of SHA-512 is not required for this purpose.

The original sensor-event record is retained by the client and replicas and is not replaced by the digest. The compact digest is used only as the consensus representation of that record, preventing the PBFT command size from increasing with the amount of sensor data.

The client includes its compact digest in a consensus request and sends it to the primary. The primary compares the client’s digest with its locally generated digest. If the digests differ, the primary does not proceed with the request. This comparison prevents the primary from initiating consensus for a sensor-event record that differs from the record held locally by the primary. If they match, the primary includes the authenticated client REQUEST in the PRE-PREPARE message and sends it to the backups. During the PRE-PREPARE phase, each backup verifies the authenticated client REQUEST and compares the client’s digest with its locally generated digest. A backup that detects a mismatch does not send a PREPARE message. Therefore, a correct backup does not endorse a request whose digest differs from the sensor-event record that it holds locally. Replicas with matching digests proceed through the PREPARE and COMMIT phases.

Each sensor group satisfies n≥3f+1 to tolerate at most *f* Byzantine replicas. A Byzantine replica may omit messages, send an incorrect digest, or send conflicting messages to different participants. Algorithm 1 specifies the behavior of correct replicas; Byzantine replicas are not assumed to follow these processing rules. Consensus messages are authenticated to detect message modification and replica impersonation. In this paper, Byzantine fault tolerance is limited to the PBFT replica group.
**Algorithm 1** Proposed Group-Based Consensus Scheme**Require:** A preconfigured PBFT replica group $G=\{r_{0},r_{1},\ldots ,r_{n-1}\}$, client *C*, shared sensor-event record *E*, and Byzantine fault bound *f*
**Ensure:** An accepted consensus result or no accepted result
  1:**Precondition:** n≥3f+1  2:**for all** participants x∈G∪{C} **do**  3:    $E_{x}^{\mathrm{norm}}\leftarrow \mathrm{NormalizeAndOrder}E_{x}$  4:    $d_{x}\leftarrow \mathrm{SHA}256E_{x}^{\mathrm{norm}}$  5:**end for**  6:Client *C* constructs an authenticated REQUEST containing $d_{C}$  7:Client *C* sends the REQUEST to primary replica *p*  8:Primary *p* verifies the authenticated client REQUEST  9:Primary *p* compares $d_{C}$ with its locally generated digest $d_{p}$10:**if** $d_{C}\neq d_{p}$ **then**11:    Primary *p* does not proceed with the request12:    **return** no accepted result13:**end if**14:Primary *p* assigns view number *v* and sequence number *s*15:Primary *p* includes the authenticated client REQUEST in PRE-PREPARE(v,s,REQUEST)16:Primary *p* sends the PRE-PREPARE message to all backups17:**for all** backup replicas $r_{i}\in G\setminus \left\{p\right\}$ **do**18:    Verify the PRE-PREPARE message and authenticated client REQUEST19:    Compare $d_{C}$ with the locally generated digest $d_{i}$20:    **if** $d_{C}\neq d_{i}$ **then**21:        Do not send a PREPARE message22:    **else**23:        Send $\mathrm{PREPARE}(v,s,d_{C},i)$ to the replica group24:    **end if**25:**end for**26:**for all** replicas $r_{i}\in G$ **do**27:    **if** a valid PRE-PREPARE and 2f matching PREPARE messages are received **then**28:        Enter the prepared state29:        Send $\mathrm{COMMIT}(v,s,d_{C},i)$ to the replica group30:    **end if**31:    **if** 2f+1 matching COMMIT messages are received **then**32:        Enter the locally committed state33:        Execute the requested operation34:        Send an authenticated REPLY message to client *C*35:    **end if**36:**end for**37:**if** client *C* receives f+1 matching authenticated replies from distinct replicas **then**38:    Accept the consensus result39:    Forward the sensor-event record associated with the accepted digest and the consensus result to the factory proxy server40:    **return** accepted consensus result41:**else**42:    Do not accept the consensus result43:    **return** no accepted result44:**end if**


Digest validation and PBFT consensus have different roles in the proposed scheme. Digest validation determines whether a correct participant holds the same normalized sensor-event record represented by the client request. PBFT then ensures that correct replicas reach a consistent processing result for the accepted request. The proposed scheme does not use PBFT to determine the physical accuracy of individual sensor data.

Algorithm 1 summarizes the digest-based group PBFT procedure. The replica group is configured before consensus according to the production process or sensor characteristics. The client and replicas organize the shared sensor data using the same predefined format and order. Only replicas whose locally generated digest matches the client-provided digest proceed with the PBFT phases.

The algorithm can be divided into four processing stages: local sensor-event preparation, request and digest validation, PBFT agreement, and client-side result acceptance.

Lines 1–5 describe local preparation before consensus. The client and replicas independently normalize their local sensor-event records and compute their compact digests. These operations are performed locally by each participant rather than by a centralized entity.

Lines 6–16 describe client request generation and primary validation. The primary verifies the authenticated request and compares the client-provided digest with its local digest before initiating PRE-PREPARE. A mismatch terminates processing of that request at the primary.

Lines 17–25 describe backup-side validation. Each correct backup independently verifies the request and compares the client digest with its locally generated digest. Only a backup with a matching digest contributes a PREPARE message.

Lines 26–36 describe the PBFT agreement procedure. Replicas that obtain the required PREPARE evidence enter the prepared state and subsequently exchange COMMIT messages. A replica executes the requested operation only after satisfying the local commit condition.

Lines 37–44 define the client-side acceptance condition. The client accepts the result only after receiving *f* + 1 matching authenticated replies from distinct replicas; otherwise, no result is accepted for the request.

In the proposed scheme, each PBFT group performs consensus independently from other groups. PRE-PREPARE, PREPARE, and COMMIT messages are exchanged only within each group. Adding a new sensor group does not increase replicas in existing groups. It also does not expand their message exchange scope.

For example, consider a replica group with four sensors, where one sensor acts as the primary and the remaining three sensors act as backups. With *n* = 4, the group tolerates *f* = 1 Byzantine replica. If the client and three correct replicas generate the same digest d, the correct replicas can proceed through PREPARE and COMMIT even when one replica does not provide a valid message. For *f* = 1, local commitment requires 2*f* + 1 = 3 matching COMMIT messages, and the client accepts the result after receiving *f* + 1 = 2 matching authenticated replies.

The prototype implemented in this paper supports standard PBFT view changes for Byzantine primary faults. If the primary provides no valid PRE-PREPARE, backups send VIEW_CHANGE messages for a new view. Invalid or recipient-specific conflicting digests are detected during verification. PRE-PREPARE omission is detected by timeout. The new primary collects required VIEW_CHANGE messages and constructs NEW_VIEW. It reproposes incomplete requests. Replicas resume PBFT in the new view. The request completes when the client receives the required number of matching replies. This procedure runs within each independent PBFT group.

### 3.4. Consensus Result Collection

After local commitment, each correct replica sends a REPLY message containing the processing result to the client. The client accepts the result after receiving f+1 matching authenticated replies from distinct replicas. Under the assumed fault bound, this threshold ensures that at least one correct replica supports the accepted result. If the required replies are not received, the client does not accept the result. After acceptance, the client forwards the sensor-event record associated with the accepted digest and the consensus result to the factory proxy server. The factory proxy server collects data delivered by each client. It then forwards the collected data to the management server. Neither server participates in PBFT consensus.

The factory proxy server does not provide global consistency or atomic ordering across replica groups. Each replica group operates as an independent consensus domain, and only results for which consensus has been completed within each group are collected by the proxy server. Accordingly, conflicts or temporal inconsistencies among results generated by different groups are not reconciled by the proposed scheme. Such cross-group coordination is outside the scope of this paper.

This separation keeps PBFT consensus within each sensor group and separates group-level agreement from factory-level result collection. The factory proxy server only aggregates results that have already been accepted by the corresponding group clients and does not affect the PBFT quorum or state of any replica group. As a result, result collection across multiple production processes does not require a common factory-wide PBFT instance.

## 4. Evaluation

### 4.1. Experimental Setup and Methods

The evaluation comprised five experiments. The experiments separated effects of group configuration and consensus input representation. They also evaluated increasing sensor counts, PBFT group sizes, and original sensor-event record sizes. The final experiment evaluated Byzantine fault handling and performance in a distributed environment.

The first experiment separated the effects of group configuration and consensus input representation. A total of 16 logical replicas was fixed. One n=16 group and four independent n=4 groups were configured. Full-data and digest inputs were applied to each group configuration. Replica-local computation costs were compared across the resulting four conditions. For the four-group condition, the computational cost was calculated for each group. The maximum was used as the representative value under assumed parallel execution. The second experiment compared computation costs as the total sensor count increased. The logical sensor count increased from 10 to 100. The original record size was fixed at 1024 B. The baseline placed all replicas in one expanding PBFT group. It used the complete sensor data as the consensus command. The proposed scheme maintained 10 replicas per group. It used the digest as the consensus command. Each role’s representative cost was multiplied by its replica count. This yielded aggregate replica-local computation cost for the complete sensor set. The third experiment examined effects of PBFT group size on individual replica workload. Group size varied across n=4,7,10,16. Replica-local computation costs were measured separately for primary and backup roles. The fourth experiment evaluated cost differences between full-data and digest inputs across record sizes. One n=7, f=2 group was used. Original record sizes were 256 B, 1 KiB, 4 KiB, 16 KiB, and 64 KiB. Primary and backup replicas processed full data from the same original records. They also processed the corresponding 32-byte SHA-256 digests.

The first four experiments were conducted on one Raspberry Pi 4 Model B Rev. 1.5. The system ran Debian GNU/Linux 13 on AArch64. The Java runtime was OpenJDK 21.0.11. Measurements included PBFT message processing, HMAC-based authentication, state transitions, callbacks, and outgoing-object serialization. Each condition was independently executed 10 times in separate JVM processes. The mean from each run was treated as one statistical sample. The 95% confidence interval for each condition mean used Student’s *t* distribution.

The fifth experiment evaluated Byzantine fault handling and consensus recovery in a distributed network. One replica was deployed on each of four Raspberry Pis. Together, they formed one n=4, f=1 PBFT group using digests as consensus input. Invalid digests were injected into backups during PREPARE and COMMIT. Recipient-specific conflicting digests and message omissions were also injected. Primary faults included invalid PRE-PREPARE digests and recipient-specific conflicting PRE-PREPARE messages. PRE-PREPARE omission was also injected into the primary. End-to-end consensus latency and throughput were measured under normal and backup-fault conditions. View-change recovery latency was measured under primary-fault conditions.

The distributed experiments were conducted using four Raspberry Pi devices connected over a network to form a single PBFT group. The normal condition and each fault condition were evaluated 10 times, with all replica processes restarted to their initial state before each run. End-to-end latency was measured over 50 requests following 20 warm-up requests, and the p50 and p95 latencies were calculated for each run. The 95% confidence intervals for latency were estimated using a run-level percentile bootstrap. Throughput was measured as the number of requests completed over 10 s with a concurrency level of 16, and its 95% confidence interval was calculated using Student’s *t* distribution. Recovery latency was measured from the initiation of a fault request through fault detection, VIEW_CHANGE, NEW_VIEW, request reproposal, and receipt of matching replies in the new view.

### 4.2. Results

Each experimental result was derived from 10 independent runs per condition. We calculated 95% confidence intervals for mean, p50, p95, and throughput estimates. Boxplots show the distribution of values across independent runs. Error bars in line graphs indicate 95% confidence intervals.

**Figure 5:**
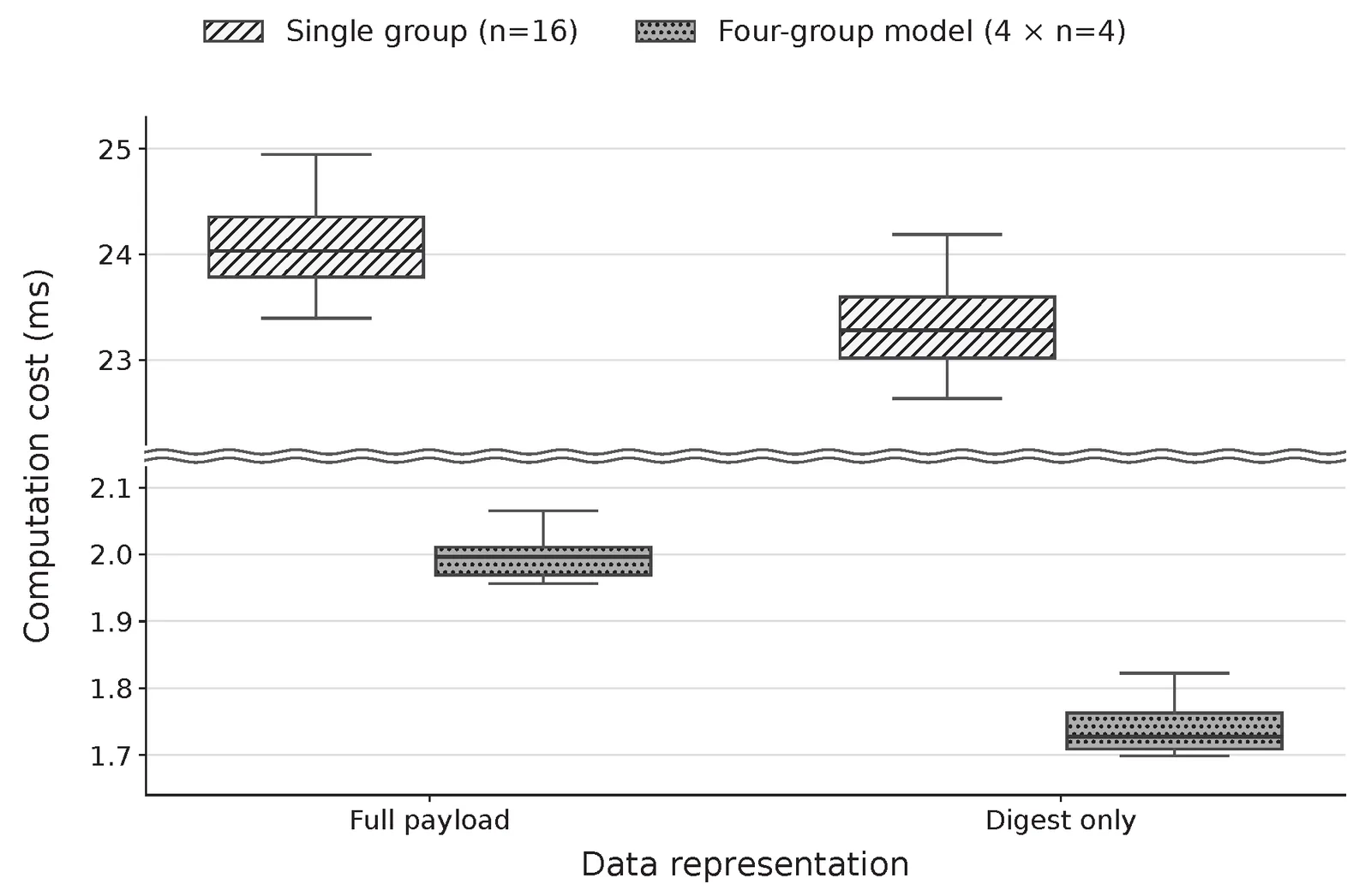
Comparison of computational cost by group configurations and data representations.

Figure 5 shows results separating group configuration and input representation across 16 logical replicas. Under full-data conditions, the mean computation cost was 24.124 ms in the single group. Its 95% CI was 23.762–24.485 ms. Across four groups, the mean was 1.996 ms with a 1.972–2.020 ms 95% CI. Under digest conditions, the corresponding means were 23.360 ms and 1.739 ms. Their 95% CIs were 22.994–23.726 ms and 1.710–1.767 ms, respectively. The four-group configuration reduced cost by 91.7% for full data and 92.6% for digests. Within identical group configurations, digests reduced cost by 3.2% in the single group. The reduction was 12.9% across four groups. Both design factors affected computation cost, but grouping produced the larger difference.

**Figure 6:**
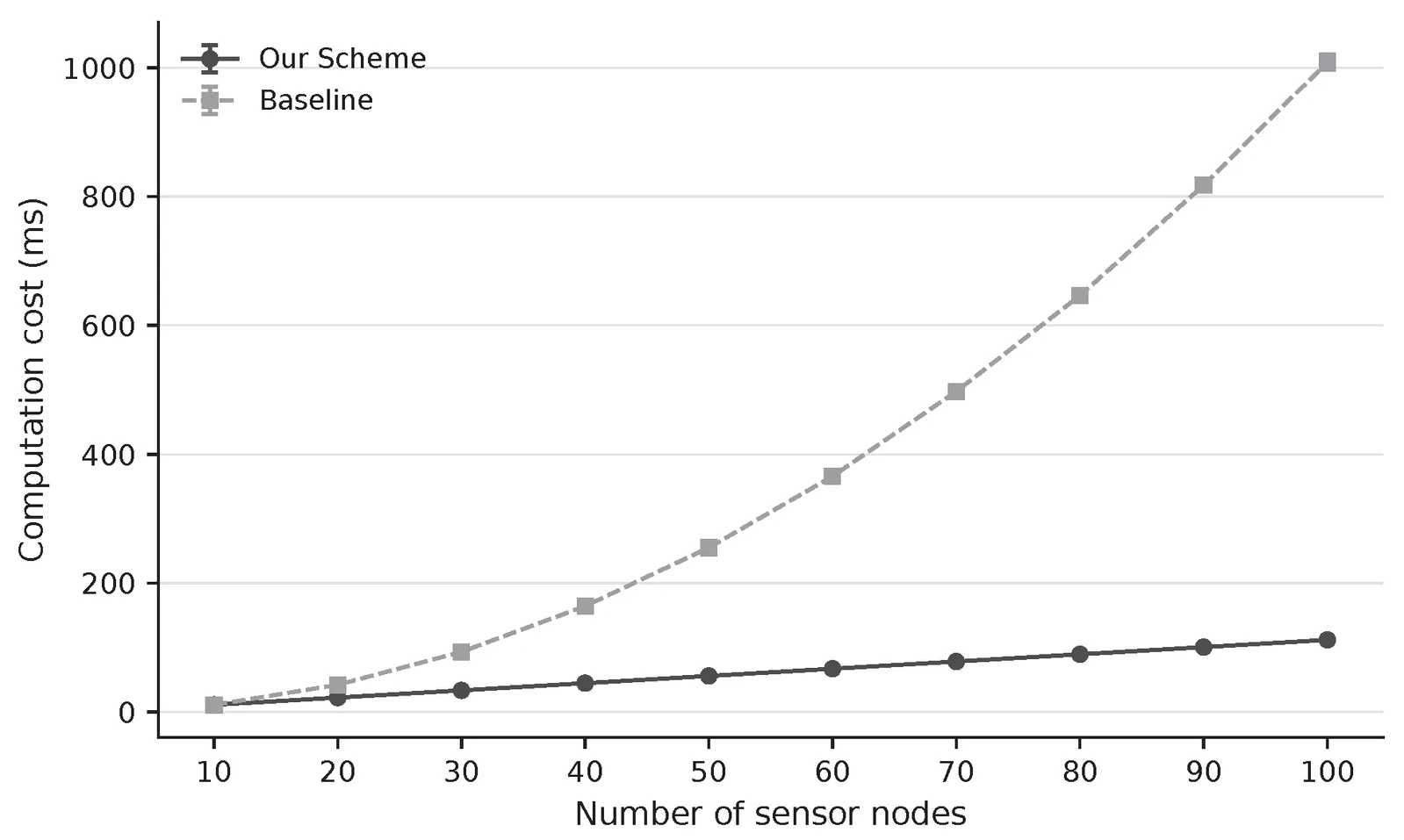
Comparison of computational cost by number of sensor nodes.

Figure 6 shows changes in aggregate replica-local computation cost across 10–100 logical sensors. At 10 sensors, mean costs were 11.097 ms for baseline and 11.198 ms for proposed scheme. The baseline increased to 255.204 ms at 50 sensors and 1008.704 ms at 100 sensors. The proposed scheme showed 55.990 ms and 111.980 ms under the same conditions, respectively. Within the evaluated range, its aggregate computation cost increased approximately linearly with sensor count. Fixed-size groups and digest inputs reduced cost growth compared with one continuously expanding PBFT group.

Let total logical replicas be *N*, fixed group size *g*, and independent groups k=N/g. We calculated inter-replica message counts for steady-state PBFT. When all *N* replicas form one group, PRE-PREPARE requires N−1 transmissions. PREPARE and COMMIT require ${\left(N-1\right)}^{2}$ and N(N−1) transmissions, respectively. The three phases total 2N(N−1) transmissions. Thus, a single *N*-replica PBFT group has $O(N^{2})$ communication complexity. For *k* independent groups of size *g*, PRE-PREPARE requires k(g−1) transmissions. PREPARE and COMMIT require $k{\left(g-1\right)}^{2}$ and kg(g−1) transmissions, respectively. The total is 2kg(g−1). Accordingly, *k* independent instances of Algorithm 1 have $O(kg^{2})$ aggregate inter-replica communication complexity. Since N=kg, this is equivalently O(Ng). This bound represents aggregate normal-case transmissions across all groups. With fixed *g*, the complexity becomes O(N).

The steady-state message counts in Table 2 theoretically explain the growth patterns of both architectures. At N=100 and g=10, the baseline required 19,800 inter-replica transmissions. The independent-group architecture required 1800 transmissions. Under the same conditions, aggregate computation costs differed by approximately 9.008 times. Message count and computation cost represent different metrics. Computation cost includes message processing, digest operations, authentication, state transitions, and serialization.

**Figure 7:**
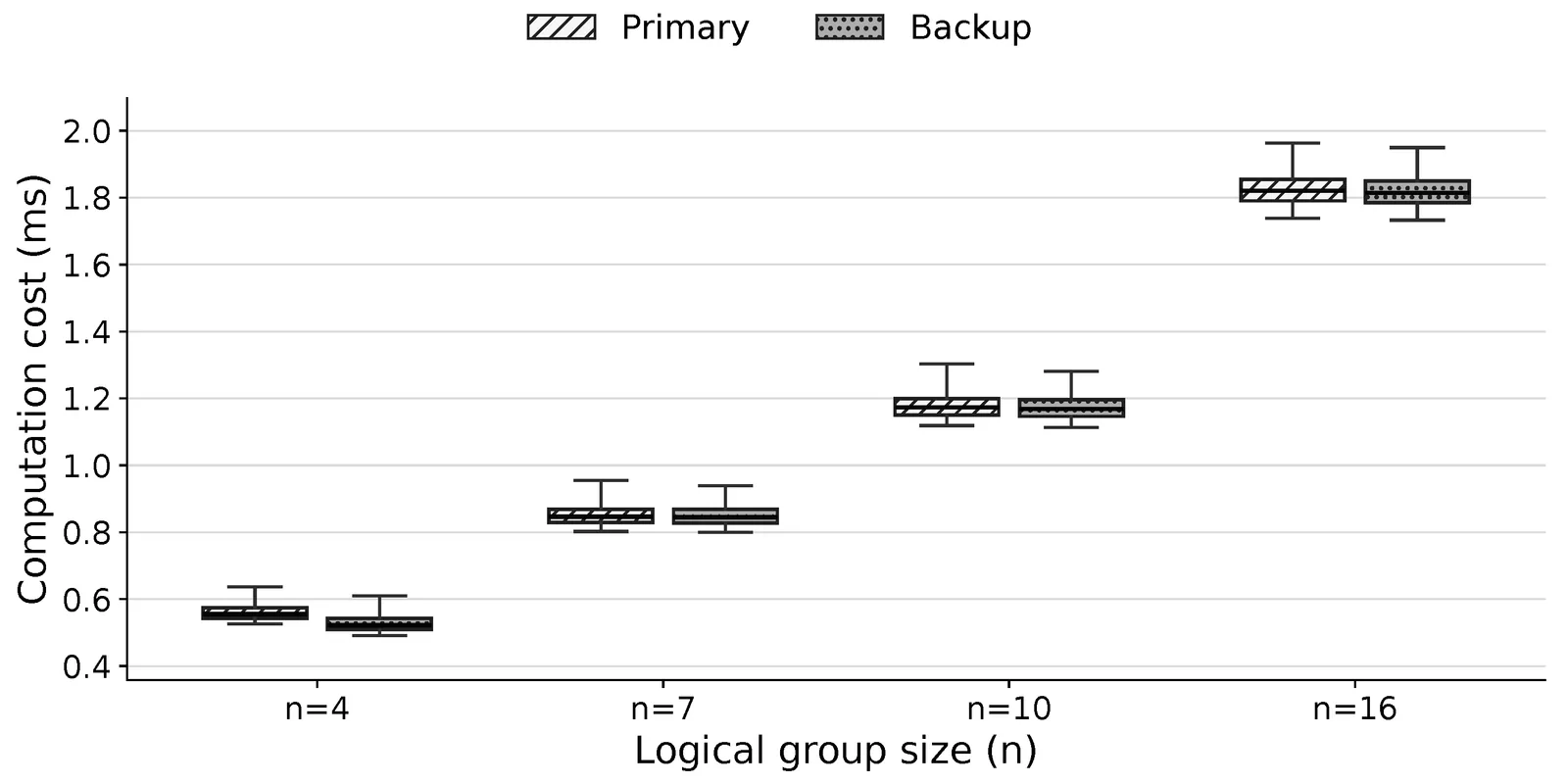
Comparison of computational costs by group size and replica role.

Figure 7 shows replica-local computation costs for primary and backup roles across PBFT group sizes. At n=4, mean costs were 0.566 ms for primary and 0.532 ms for backup. The corresponding 95% CIs were 0.560–0.572 ms and 0.527–0.537 ms. At n=7, mean costs were 0.859 ms and 0.857 ms, respectively. At n=10, they were 1.188 ms and 1.182 ms, respectively. At n=16, primary cost increased to 1.838 ms. Its 95% CI was 1.820–1.857 ms. Backup cost increased to 1.832 ms, with a 1.813–1.851 ms 95% CI. Both roles showed similar growth across all evaluated group sizes. These results show the importance of limiting group size to control replica-local PBFT workload.

**Figure 8:**
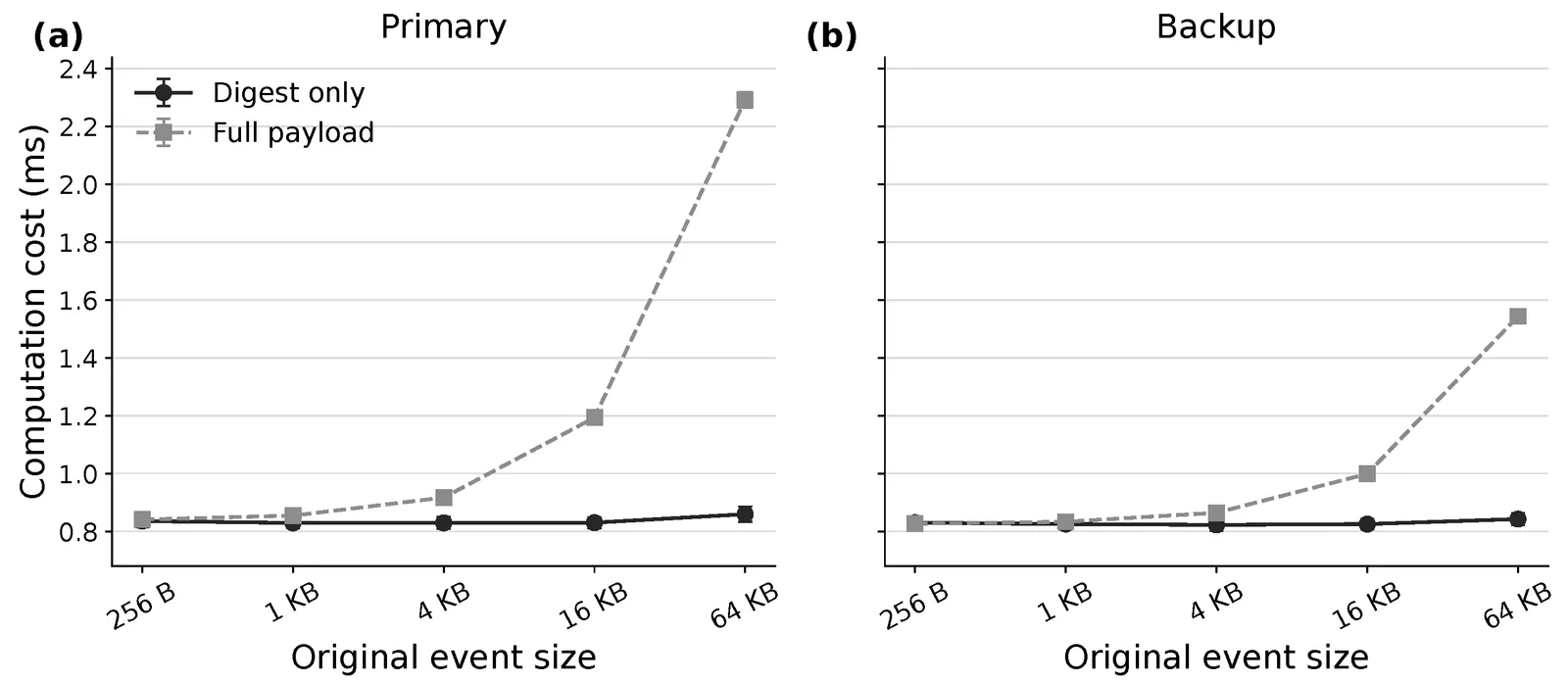
Comparison of computational costs by event record size and data representation. (**a**) Primary node; (**b**) Backup node.

Figure 8 shows computation costs for full-data and digest inputs across original record sizes. (a) presents primary costs and (b) presents backup costs. At 256 B, primary costs were 0.841 ms and 0.836 ms for full-data and digest inputs, respectively. Backup costs were similarly 0.826 ms and 0.830 ms, respectively. At 1 KiB, the difference between both inputs remained small. At 4 KiB, primary costs were 0.917 ms and 0.829 ms, respectively. Their 95% CIs were 0.897–0.937 ms and 0.809–0.849 ms. Backup costs were 0.864 ms and 0.822 ms, respectively. Their 95% CIs were 0.845–0.883 ms and 0.802–0.843 ms.

Larger original records at 16 KiB and 64 KiB produced clearer full-data cost increases. At 16 KiB, full-data costs were 1.193 ms for primary and 0.999 ms for backup. Digest costs were 0.830 ms and 0.825 ms, respectively. At 64 KiB, primary full-data cost was 2.292 ms. Its 95% CI was 2.265–2.318 ms. The corresponding digest cost was 0.859 ms. Its 95% CI was 0.833–0.885 ms. Backup costs were 1.544 ms and 0.843 ms for full-data and digest inputs, respectively. Their 95% CIs were 1.526–1.561 ms and 0.823–0.862 ms. At 64 KiB, full-data costs exceeded digest costs by 2.667 times for primary. For backup, full-data costs were 1.832 times higher. Across 256 B–64 KiB, digest costs changed less with increasing record size than full-data costs. Both input representations used identical PBFT group configurations and message transmission counts. This difference reflects input representation and resulting replica-local processing, not fewer messages.

**Figure 9:**
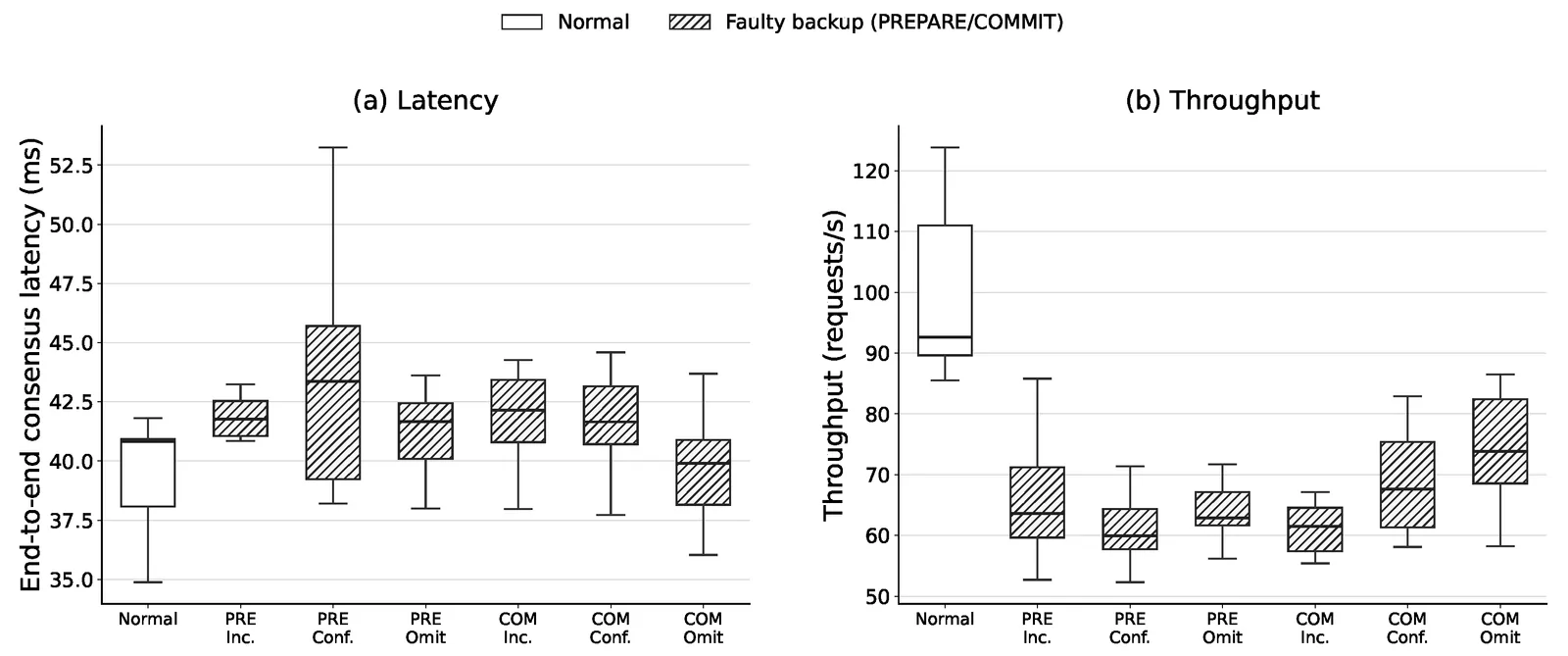
Comparison of consensus latency and throughput by Byzantine backup fault condition. (**a**) Latency; (**b**) Throughput.

Figure 9 shows end-to-end consensus latency and throughput under normal and six backup-fault conditions. (a) shows end-to-end consensus latency and (b) shows throughput. In the distributed environment, each intended backup fault occurred in all 10 independent runs. Invalid or recipient-specific conflicting PREPARE and COMMIT messages carried the faulty replica’s HMAC. PBFT message verification rejected these messages. Under omission faults, the remaining correct replicas formed a quorum. Consensus completed in all 60 runs, with zero recorded safety violations.

Under normal conditions, p50 was 40.82 ms (95% CI: 37.47–40.93 ms). Across fault conditions, p50 ranged from 39.90 to 43.37 ms. Under normal conditions, p95 was 61.13 ms (95% CI: 58.42–63.83 ms). Across fault conditions, p95 ranged from 62.71 to 69.99 ms. Changes in p50 were limited, while fault-condition p95 estimates exceeded the normal estimate. Mean throughput under normal conditions was 99.32 requests/s (95% CI: 89.41–109.23 requests/s). Backup-fault throughput ranged from 58.14 to 74.38 requests/s. All point estimates were lower than the normal-condition estimate. The lowest throughput was 58.14 requests/s under the invalid COMMIT-digest condition. Its 95% CI was 45.27–71.01 requests/s. COMMIT omission produced the highest fault-condition throughput at 74.38 requests/s. Its 95% CI was 67.42–81.34 requests/s. Consensus remained intact despite a Byzantine backup. However, fault conditions showed performance costs in p95 latency and throughput.

**Figure 10:**
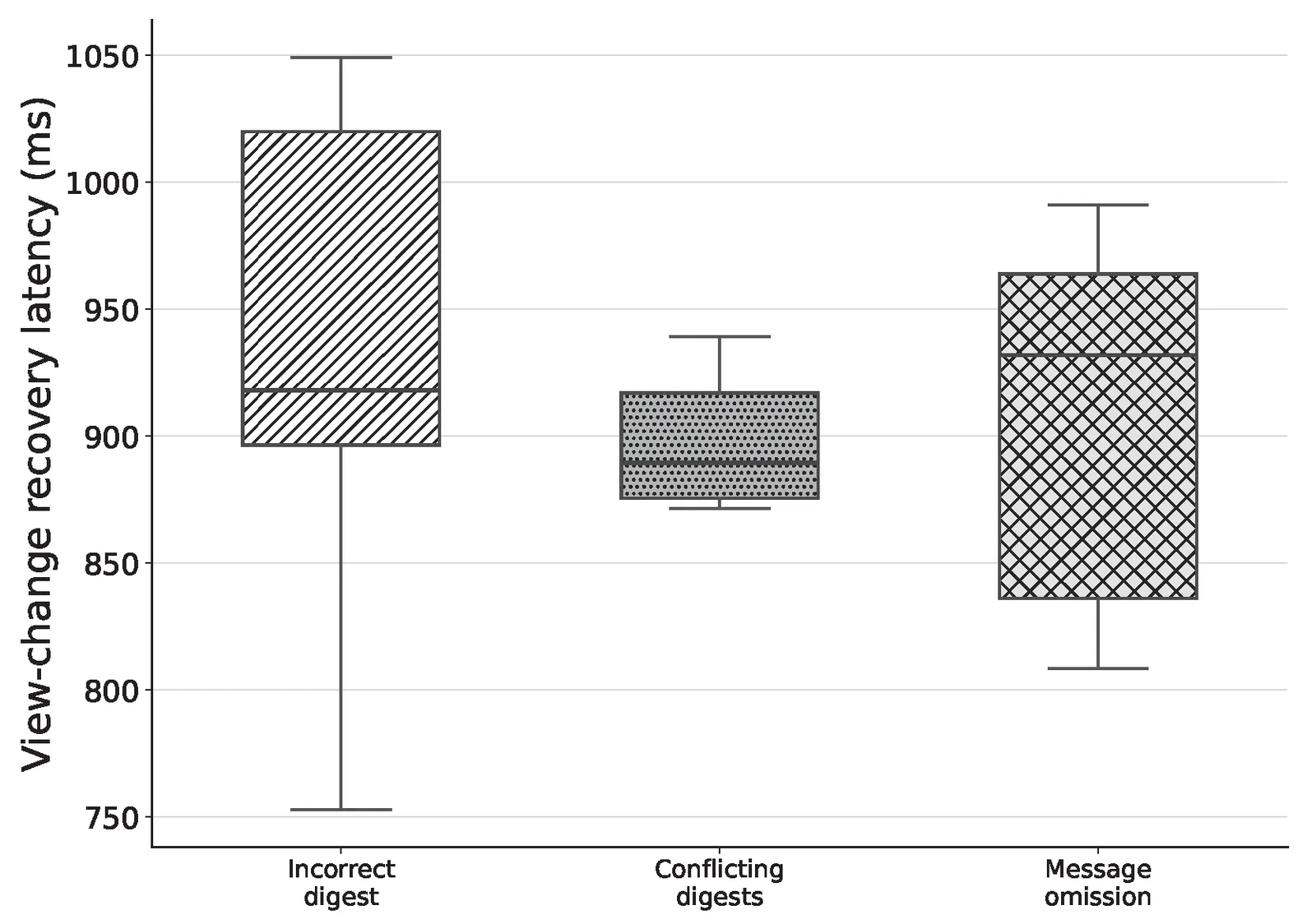
Comparison of view-change recovery latency by Byzantine primary fault condition.

Figure 10 shows recovery results through view changes after Byzantine primary PRE-PREPARE faults. Under invalid PRE-PREPARE digests, PBFT message verification rejected the abnormal PRE-PREPARE. For recipient-specific conflicting digests, PBFT message verification also rejected the abnormal PRE-PREPARE. Under PRE-PREPARE omission, the timeout detected the omission. In all three conditions, VIEW_CHANGE and NEW_VIEW occurred in each of 10 independent runs. Final consensus completed after the new primary reproposed the request. Across all 30 independent runs, zero safety violations were recorded.

Median view-change recovery latency was 918.26 ms for an invalid PRE-PREPARE digest. It was 889.50 ms for conflicting digests and 931.95 ms for PRE-PREPARE omission. Recovery from fault initiation to final matching replies required approximately 0.89–0.93 s. This interval included detection, VIEW_CHANGE, NEW_VIEW, and request reproposal. Successful recovery under all three faults shows that processing can resume in a new view. This recovery occurs even after a Byzantine primary disrupts consensus.

These results show that independent groups and digest inputs reduce PBFT processing costs differently. Independent groups limit the number of replicas participating in consensus. Digest inputs mitigate processing-cost growth as original records become larger. Consensus is also completed with Byzantine backups in the distributed environment. After Byzantine primary faults, the view changes successfully restored the consensus. However, fault conditions introduced additional performance costs, including reduced throughput.

## 5. Discussion

The clearest difference appeared in the scope of replicas participating in consensus. With identical input representation, four independent n=4 groups had lower replica-local computation cost. This cost was lower than that of a single n=16 group. Under identical group configurations, digest inputs incurred lower costs than full-data inputs. Figure 5 shows independent groups and digest inputs as complementary designs reducing different overheads.

Role-specific measurements across group sizes support this interpretation at the individual-replica level. Figure 7 shows increasing local processing costs for both primary and backup roles. Both roles exhibited similar growth as the group size increased. Fixed-size groups separate factory-wide sensor counts from each replica’s processing scope. However, the 10-node group is an evaluation setting in this paper. Actual group sizes should consider process coupling, data-sharing scope, fault tolerance, and device resources.

The increase in individual-replica cost also aligns with aggregate results across total sensor counts. Table 2 shows that steady-state inter-replica message counts grow as $O(N^{2})$ in a single group. They grow as O(N) with fixed-size independent groups. At N=100, theoretical message counts differed by a factor of 11. Figure 6 shows approximately a ninefold difference in aggregate computation cost. The ratios differ because computation cost includes several additional processing components. These include digest processing, message authentication, state transitions, and serialization.

The effect of digest input became clearer as original sensor-event records increased in size. Figure 8 shows similar costs for both input methods with small records. However, full-data processing costs increased faster as original record size increased. Both conditions used identical group configurations and message counts. This difference arises from consensus input representation and resulting internal processing.

Results from the distributed environment show both fault tolerance and associated performance costs. Invalid or conflicting messages from Byzantine backups were rejected during verification. When messages were omitted, correct replicas formed the required quorum. All evaluated backup-fault conditions in Figure 9 completed consensus successfully. However, p95 latency and throughput point estimates differed from normal conditions. Consensus also resumed after Byzantine primary faults through VIEW_CHANGE and NEW_VIEW. Figure 10 presents the corresponding recovery latency. These results show that consensus can persist and recover under fault conditions. Such fault handling, however, introduces additional processing costs.

The applicability of the proposed scheme should consider its independent-group design. Each group performs consensus independently from other groups. Processes requiring atomic ordering or global consistency across groups need additional coordination. The current distributed evaluation also covers only one n=4, f=1 group. Future evaluations should include multiple physical groups and heterogeneous devices. This expansion would better reflect practical deployment characteristics. Network conditions, memory usage, and energy consumption should also be analyzed. Such analysis can further clarify group-size selection for different deployment environments.

## 6. Conclusions

Smart factories rely on IIoT sensor data for equipment monitoring and automated process control. However, faulty or Byzantine sensors can cause replicas to process inconsistent sensor-event records. Individual message authentication alone does not ensure that non-faulty replicas reach the same processing result. To address this problem, this paper proposes a group-based consensus scheme in which independent replica groups execute PBFT using compact event digests. Sensors are grouped according to production processes or sensor characteristics, and each group performs consensus independently. Each client forwards the sensor-event record associated with the accepted digest and the consensus result to the factory proxy server. This architecture avoids placing all factory sensors in one expanding PBFT group and allows new production lines to form separate groups without modifying existing groups.

Experimental results show that the designs proposed in this paper reduce different IIoT processing burdens. Independent groups restrict each replica’s consensus participation to its group as factory sensor counts increase. They also allow new production lines without expanding existing groups. Fixed-length digests decouple consensus input size from original sensor-event record size. This reduces replica-local processing overhead as sensor data volume increases. In the distributed environment, consensus remained intact despite invalid, conflicting, or omitted messages. After Byzantine primary faults, view changes allowed consensus to resume. These properties demonstrate the proposed scheme’s benefits for resource-constrained smart factories. It controls consensus scope and input size while tolerating Byzantine faults.

Future work should evaluate concurrent execution across multiple physical replica groups. It should also consider heterogeneous devices, network delay, packet loss, memory, and energy consumption. Inter-group coordination should also address result conflicts and ordering across independent groups.

## Figures and Tables

**Table 1:** Comparative analysis of recent BFT studies.

Type	Ref.	Objective	Description	Strength	Limitation
Hierarchical/Group-Based BFT	[12]	Scale IoT consensus networks	Partitions proxy devices into domains with two-level multi-primary consensus	Reduces communication growth and improves throughput in simulations	No physical IoT devices
	[13]	Scale leader-based consensus systems	Divides replicas into equal-sized groups with leader vote aggregation	Reduces leader bottlenecks and improves throughput at larger scales	Uses cloud instances rather than physical IoT devices
	[14]	Scale PBFT for large blockchain networks	Uses a multi-layer architecture whose leaders aggregate member replies	Analyzes communication complexity and fault thresholds	No peer-to-peer execution
	[19]	Process heterogeneous IoT data	Separates mining groups and a packaging chain across two blockchains	Lowers consensus time and improves throughput over PBFT	Execution platform and network configuration were not reported
Committee-Based BFT	[20]	Support vehicular blockchain consensus	Uses reputation to select a leader and secondary nodes	Demonstrates performance on a physical vehicular testbed	Uses one vehicular testbed configuration
Resource-Constrained BFT	[21]	Execute PBFT-based replication on highly constrained devices	Implements bounded state-machine replication on four ESP32-C3 replicas over physical Wi-Fi	Demonstrates PBFT execution, memory use, and performance on embedded hardware	Reports network-inclusive service metrics without isolating local computation
Performance and Reliability Analysis	[22]	Analyze PBFT ordering capacity for distributed IoT systems	Models bandwidth reservation and queueing for multiple orderers	Relates delay, request queues, and geographic range to system capacity	Uses analytical modeling and simulation without physical execution
Asynchronous BFT	[23]	Improve asynchronous consensus for IoT blockchains	Combines order-first consensus, missing-transaction recovery, threshold signatures, and producer reputation	Evaluates virtual nodes under emulated network delays	Uses server-based virtual machines rather than physical IoT devices

**Table 2:** Comparison of message transmissions by group configurations.

Architecture	PRE-PREPARE	PREPARE	COMMIT	Total
Single group, size *N*	N−1	${\left(N-1\right)}^{2}$	N(N−1)	2N(N−1)
*k* independent groups, size *g*	k(g−1)	$k{\left(g-1\right)}^{2}$	kg(g−1)	2N(g−1)

## Data Availability

The data presented in this study are available on request from the corresponding author.
